# Who Should Address Healthcare Institutional Betrayal and How? An Experimental Study of Repair Strategies

**DOI:** 10.3390/healthcare14152318

**Published:** 2026-08-01

**Authors:** Fallon J. Richie, Jennifer Langhinrichsen-Rohling, Chloe Gilmore, Bridget N. Jules, Daniel T. Dickie

**Affiliations:** 1 Psychology Service, Battle Creek VA Medical Center, Battle Creek, MI 49037, USA; 2 Department of Psychological Science, UNC Charlotte, Charlotte, NC 28223, USA; jlanghin@charlotte.edu (J.L.-R.); cwoodlin@charlotte.edu (C.G.); bjules1@charlotte.edu (B.N.J.); ddickie@charlotte.edu (D.T.D.)

**Keywords:** healthcare institutional betrayal, repair, healthcare avoidance, trust, healthcare expectancies, experiment

## Abstract

**Highlights:**

**What are the main findings?**

- Healthcare system stakeholders are uniquely positioned to provide repair to patients harmed by acts of healthcare institutional betrayal.
- Using an experimental design, two distinct repair acts (empathic apology by the healthcare provider; administrator-initiated organizational change) were shown to be particularly effective in reducing residual healthcare institutional betrayal and restoring healthcare team trust.
- All tested repair acts helped to restore positive expectations for future healthcare.
- Organizational change, a type of institutional courage, was particularly effective in reducing anticipated avoidance and decreasing negative expectations for future healthcare encounters after a problematic healthcare encounter.

**What are the implications of the main findings?**

- Training healthcare system stakeholders to assess and address patients’ pre-existing healthcare-related institutional betrayal may improve patient engagement and generate more positive health outcomes.

**Abstract:**

**Background/Objectives:** Healthcare institutional betrayal (HIB) is a betrayal trauma that occurs when a healthcare organization or system perpetrates acts of wrongdoing against or fails to act to protect an individual who depends upon that system for care and services. Known consequences of experiencing acts of HIB include increased healthcare provider and system distrust and anticipated healthcare avoidance, highlighting the public health implications of unaddressed HIB. Little is known about actions that healthcare stakeholders can take to repair HIB. Thus, this study experimentally tested the effects of receiving one of two reparative actions following a healthcare encounter containing HIB (empathic apology vs. organizational change) performed by one of two healthcare system stakeholders: healthcare provider or system administrator. **Methods:** Residual HIB perceptions, trust, expectations for future healthcare, and intentions to avoid future care were assessed post-repair conditions. Initially, undergraduate participants (*N* = 198) were asked to imagine themselves experiencing a common healthcare scenario which included HIB. After post-HIB encounter baseline measurements, participants were then randomly assigned to one of four conditions (three with HIB repair actions vs. one control). Participants receiving any type of HIB repair reported significantly lower residual HIB, increased positive expectations for future healthcare, and greater trust in healthcare post-repair, with effect sizes ranging from small to large. **Results:** Generally, two HIB repair conditions (organizational change to repair HIB and healthcare provider apology repair) outperformed the healthcare administrator apology condition; all repair conditions outperformed the control condition. **Conclusions:** Our finding that specific actions can facilitate post-HIB recovery is clinically meaningful. Medical professionals and healthcare administrators need to address patients’ past negative experiences with healthcare and take action to repair pre-existing HIB to improve patients’ ongoing and future healthcare experiences.

## 1. Introduction

Institutional betrayal [1] occurs when institutions, such as universities, healthcare systems, the armed services, and religious organizations are responsible for perpetrating wrongdoings against individuals who are dependent on these systems for care and services. When these wrongdoings (through acts of omission or commission) occur within a healthcare system or via a healthcare encounter, they are labeled as healthcare institutional betrayal (HIB) [2]. For example, a healthcare system may dismiss a patient’s concerns, fail to provide best-practice care, or fail to respond adequately to patients’ complex medical needs, thereby betraying the patient’s trust and reducing their sense of safety. Experiencing at least one act of HIB is common among college students reporting on their worst healthcare experience [3,4,5] as well as among patients coping with chronic health conditions [6,7].

Experiencing acts of HIB during a problematic healthcare encounter has been linked to two critical and deleterious patient outcomes: anticipated healthcare avoidance or disengagement, and reduced trust in healthcare organizations and providers [3,8]. Post-HIB disengagement with healthcare can have serious public health implications. For example, avoiding or choosing not to utilize healthcare services may result in delayed diagnoses or worsening of chronic disease [9,10]. Conversely, research indicates that routine utilization of preventative care (e.g., engagement in breast cancer screenings and routine immunizations) is associated with better health and large healthcare savings across the system [11].

Reduced trust of healthcare system stakeholders, providers, staff, and administrators, has also been shown to be problematic. For example, a recent scoping review of the healthcare trust and mistrust literature indicates that patient trust influences cooperation and compliance with medical instructions and healthcare directives, which are critical steps toward health [12]. Importantly, institutional factors, including transparent communication, quality of services, and perceived fairness, drive the experience of trust for patients. Trust thus functions as a vital link between the experience of healthcare institutional betrayal and patient health outcomes.

It is essential to develop healthcare policies and practices that can interrupt and repair healthcare institutional betrayal [13]. Yet, to date, very little is known about the effectiveness of corrective actions that can be taken by various healthcare system stakeholders to repair HIB, maintain patient engagement, and restore trust between the individual and their healthcare institution. Understanding what actions are needed, and by whom, after a patient has experienced one or more healthcare encounters with HIB may illuminate directions for best-practice medical care and offer ways to facilitate patient healing after experiencing HIB. Thus, the goal of this research was to use an experimental design to test the effectiveness of two distinct repair strategies when utilized either by a healthcare provider or a healthcare system administrator after the experience of HIB in a routine patient healthcare encounter.

### 1.1. Theoretical Considerations

**Figure 1:**
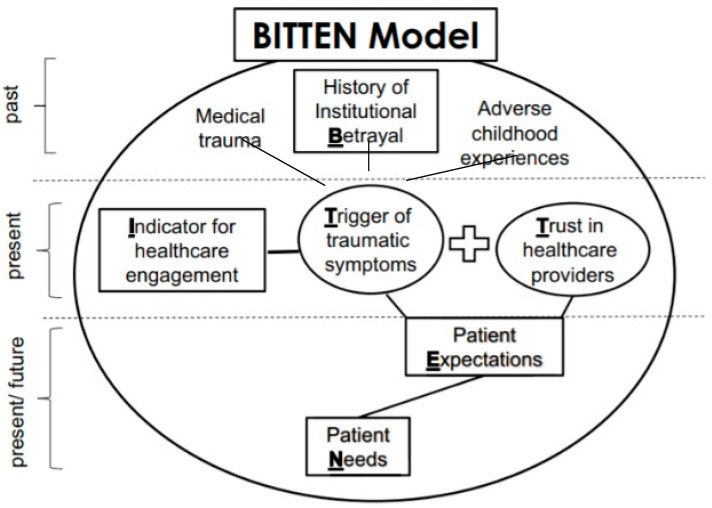
The role of healthcare institutional betrayal in the BITTEN model. Three time periods are depicted in the model. Because they influence each other, dotted lines separate the time periods. Constructs that are theoretically connected and are expected to unfold over time are linked through a solid line. Trauma symptoms that are triggered and patient trust in their healthcare provider are co-occurring in the healthcare encounter in ways that can exacerbate each other which is depicted with a plus sign.

Several existing theoretical frameworks highlight the need for repair after patient experiences of HIB. The first framework, BITTEN, describes a pathway from past experiences of HIB through the patient’s current healthcare experiences and onto their anticipated future interactions with healthcare systems. Specifically, the BITTEN framework postulates the following: A **B**etrayal history by a healthcare-related institution (i.e., pre-existing HIB), can be activated in the context of a current **I**ndicator for healthcare engagement. When activated, unresolved HIB can trigger **T**rauma symptoms, reduce **T**rust in current healthcare providers, and lead to different patient **E**xpectations and **N**eeds in both the current and in future healthcare encounters [14] (Figure 1).

An underlying assumption of the BITTEN model is that past occurrences of HIB are typically unresolved and likely have not been assessed nor addressed in the current medical encounter. The experience of HIB may have even occurred when interacting with a different healthcare team and thus may be unknown to the current provider. Yet, repair is an ongoing patient need post-HIB, particularly when the patient is seeking care in a similar situation or for the same condition as they were when the acts of HIB occurred, as these encounters may be particularly triggering.

Importantly, the BITTEN framework also aligns with the national push for trauma-informed care (TIC), which highlights the need for healthcare stakeholders to recognize and respond to patient needs and trauma symptoms with safety, transparency, and empowering choices [14]. Patients’ needs as depicted in TIC and as described in the BITTEN framework are “what the patient requires in order to be cared for in a competent and holistic manner, consisting of both physical and psychosocial needs” [14]. To fulfill those needs, responsibility for repair from HIB often falls to a medical institution’s frontline healthcare providers or their staff. To date, little, if any, research has examined outcomes related to different approaches to HIB repair, including the effects of offering the patient healthcare system-level or organizational change. The impact of experiencing different types of repair on patients’ residual HIB, anticipated avoidance, and healthcare-related trust constitutes the primary focus of the current study. The BITTEN model also proposes that HIB changes the patient’s expectations for future encounters (e.g., greater negative expectations, fewer positive expectations). Consequently, the degree to which various acts of repair, undertaken by different stakeholders, serve to restore patients’ positive expectations and ameliorate negative expectations is also considered in the current research.

A second model underlying this study’s purpose draws from betrayal trauma theory. This model emphasizes the need for a healthcare institution and its members to embrace institutional courage, particularly when faced with knowledge of patient institutional betrayal. Institutional Courage has been described as “accountability, transparency, actively seeking justice, and making reparations where needed” [15,16]. The delineation of institutional courage as the main antidote to institutional betrayal per betrayal trauma theory highlights the foundational need for medical institutions to *repair* their relationship with individuals they have harmed by being accountable for, apologizing to, and bearing witness to injustices the system and its representatives may have perpetuated. In this model, both institutional acts of repair and acknowledgement of harm are ways that institutions can exemplify courage. In keeping with this tenet, recent work by Kähkönen and colleagues (2021) [17] indicates that HIB repair must go beyond any of the specific individuals associated with committing that transgression (e.g., the patient needs more than an apology from the offender or provider). Instead, these authors suggest that organizational reform (e.g., changing organizational policies, conducting open investigations, institutional penance) is needed for HIB repair, as HIB, by definition, is a transgression that has occurred at the system level. The current study was specifically designed to test the supposition made by Kähkönen and colleagues (2021) [17] through the utilization of an experimental design that included organizational change as one potential mechanism of repair for HIB.

### 1.2. Design of the Present Study

In summary, this study examined the effects of one of two different healthcare stakeholders (healthcare provider or a healthcare system administrator) initiating one of two specific reparative actions (empathic apology or organizational change) on participants’ perceptions of HIB repair and related constructs (i.e., residual healthcare institutional betrayal, trust in the healthcare team and system) after patients had imagined themselves experiencing a routine healthcare encounter that included multiple acts of HIB. Two additional patient-related outcomes were included in the current study based on the BITTEN model: negative expectations for future healthcare and positive expectations for future healthcare.

Study aims were addressed through random assignment of participants to one of four experimental repair conditions after they experienced HIB: (1) healthcare provider apology (i.e., the healthcare provider discusses the patient’s HIB, empathizes, and apologizes in an effort to address HIB and improve the interpersonal relationship), (2) healthcare administrator apology (i.e., the healthcare administrator discusses the patient’s HIB, empathizes, and apologizes), (3) organizational change HIB repair (i.e., a healthcare system representative communicates that organizational-level change will ensue due to the patient’s experience of HIB), and (4) no repair (control condition).

Hypotheses were as follows: (1) participants assigned to vignettes with any HIB repair behaviors would report greater perceived repair (a manipulation check) than those assigned to the no repair control condition, (2) participants receiving any act of repair would report decreased residual HIB, reduced anticipated healthcare avoidance and increased levels of restored trust in the healthcare system post-HIB, and (3) participants assigned to any of the three HIB repair conditions would also report higher positive expectations for future healthcare encounters and lower negative expectations for future healthcare encounters.

In keeping with theory, a priori, it was further expected that participants would report the best outcomes (greatest reductions in residual HIB, anticipated avoidance, negative expectation and most restored trust and positive expectations) in response to the organizational change HIB repair condition as that type of repair would serve to acknowledge the patient’s own HIB, while simultaneously increasing the likelihood of preventing future occurrences of HIB for both self and others, thus, theoretically earning patient respect by demonstrating institutional courage.

## 2. Materials and Methods

### 2.1. Participants

An a priori power analysis conducted using G*Power 3 [18] estimated that 232 participants would be needed to reliably detect differences among four different groups with a medium effect size (*f* = 0.25), α = 0.05, and power of 0.90. Given this analysis, 245 participants were recruited for and participated in this study. However, after removing responses from participants who failed any of the three embedded validity checks, duplicate participants, participants who completed very little of the survey prior to dropping out, and responses from seven non-traditional students who were age outliers and likely had substantially more experience with the healthcare system than the average participant, 191 participants were retained for data analysis.

All 191 participants were undergraduate students from a large public university located within the Southeastern United States who were choosing to participate in this study as one option to fulfill an introductory psychology course requirement. Full demographics on the final sample are depicted in Table 1.

Participants ranged in age from 17 to 27, with a mean age of 19.87 years (*SD* = 2.0 years). Most participants self-identified as women (57%) and as heterosexual (79%). Just over half the sample (51%) self-identified as White; participants also reported identifying as Black (20%), Asian (9%), Other/Latino (4.7%) and multi-racial (9%) among other identities. Thirty-one students identified their ethnicity as Hispanic (*n* = 31). Almost all participants indicated that they received the majority of their healthcare within the United States (97%).

All participants were recruited online via the psychology department’s SONA research system. A brief description of the study was given in the recruitment phase and then all participants read and responded affirmatively to an informed consent document prior to study initiation. Participants received either extra credit or course credit upon study completion; other options to complete course requirements were readily available. Participants could choose not to answer any or all questions. IRB approval was obtained before this study was conducted, and ethical procedures were followed throughout.

### 2.2. Procedure

At the outset, all participants were randomly assigned to read one of two standard healthcare scenarios. Both depicted multiple acts of HIB occurring within a primary care visit that was initiated by the participant. Full vignettes are available from these authors upon request. The two scenarios were identical except for the presenting medical problem; both problems were chosen to be common concerns of college students: persistent headaches (*n* = 90) or unexplained abdominal pain (*n* = 101). A pilot study of these stimuli confirmed the number of HIB acts needed so these scenarios would be perceived as both realistic and also healthcare institution-betraying by participants. Each scenario included the same six HIB acts that were drawn from Smith’s (2017) [2] Healthcare Institutional Betrayal Questionnaire (IBQ-H). These were (1) minimizing the patient’s concerns, (2) denying the patient’s experience, (3) not taking steps to prevent unpleasant healthcare experiences, (4) making it difficult to report concerns, (5) experiencing a lack of communication between providers, and (6) seeing that the system may have created an environment in which continuing to seek care was difficult [2]. After reading and imagining themselves in their assigned healthcare scenario, participants completed measures assessing their perceptions of whether HIB occurred in the encounter they just read about, the degree to which they were immersed in the scenario, whether it was realistic, and if they perceived it as something that could happen to them (serving as HIB healthcare scenario manipulation checks). Baseline (post-HIB) assessments of their level of trust in the depicted healthcare team and the associated healthcare system, given the scenario they had experienced, were also assessed. Finally, all participants were asked about their positive and negative expectations for future healthcare encounters after experiencing their HIB scenario.

After completing these post-HIB healthcare scenario measures, all participants were again randomly assigned to one of the four experimental post-HIB repair scenarios: (1) healthcare provider apology repair (i.e., discuss the HIB, empathize, and apologize), (2) healthcare administrator apology repair (i.e., the system representative discusses the HIB, empathizes, and apologizes), (3) organizational change repair (i.e., system representative explains that the patient’s HIB experience has prompted organizational-level change), and (4) no repair (control condition). After reading their randomly assigned repair vignette, participants rated their residual HIB, current trust in this healthcare team and system, their anticipated healthcare avoidance/disengagement, and their expectations for future healthcare encounters (positive and negative). They also rated their perceptions of whether they experienced repair via what was offered to them (i.e., these six questions served as the HIB repair manipulation check). To conclude the study, participants answered demographic questions.

### 2.3. Measures

#### 2.3.1. Healthcare Institutional Betrayal: Institutional Betrayal Questionnaire—Medical Systems (IBQ-MS)

The IBQ-MS is a 42-item scale measuring IB within the medical system [19]. It consists of three components—negative cognitive–affective patient reactions, doctor- and system-level actions, and system-level reactions to reports of negative healthcare experiences. Items are measured on a five-point Likert scale (from “never” to “almost always” for behavioral items and “not at all” to “extremely” for emotional response items). Example items from the IBQ-MS include: “Made you feel humiliated”, “Medical providers do not listen to patients’ concerns”, and “The medical system denied your experiences in some way”. In the current study, the coefficient alpha for this measure was excellent at 0.97. The IBQ-MS was administered twice, once after the initial HIB scenario to document that HIB was experienced in the imagined scenario and again after the HIB repair or no repair condition to determine the amount of HIB remaining after receiving repair or not (residual HIB).

#### 2.3.2. Healthcare Institutional Betrayal Repair Manipulation Check

A pilot study was conducted to test the efficacy of six HIB repair questions that were used to ensure that participants experienced repair in the three HIB repair conditions (HIB Repair) [20]. Factor analysis indicated all six items loaded onto a single factor with excellent internal consistency. Consequently, all were retained and utilized in the current study: (1) “After your last interaction, to what degree do you believe that the provider’s behavior facilitated restoring your trust in them?” (2) “After your last interaction, to what degree do you believe that the provider’s behavior facilitated restoring your trust in that healthcare clinic?” (3) “After your last interaction, to what degree do you believe that the provider’s behavior facilitated restoring your trust in the healthcare system as a whole?” (4) “After your last interaction, to what degree did their behavior sufficiently repair your relationship with the provider you saw?” (5) “After your last interaction, to what degree did their behavior sufficiently repair your relationship with the clinic where you were receiving services?” and (6) “After your last interaction, to what degree did their behavior sufficiently repair your relationship with the healthcare system as a whole?” Responses were coded on a scale from 1 “not at all” to 5, “extremely”. A total score was computed by summing up all six items. A second factor analysis of these six items in the current sample indicated that all items again loaded onto a single factor, indicating a cohesive construct, with excellent internal consistency (*α* = 0.93). The HIB Repair measure was administered only once, after participants experienced one of the four repair conditions.

#### 2.3.3. Wake Forest Physician Trust Scale

This is a 10-item scale which assesses an individual’s trust in their physician. For this study, items were reworded slightly to address trust in the healthcare team depicted in the scenarios [21]. Item responses are scored on a five-point Likert scale from “strongly agree” to “strongly disagree”. An example item includes “Your healthcare team is extremely thorough and careful”. The coefficient alpha for this measure was excellent (*α =* 0.94) in the current study. The Wake Forest Physician Trust Scale was also administered twice, once after the initial HIB scenario and again after the HIB repair or no repair condition vignette.

#### 2.3.4. Expectations for Healthcare

The Negative Expectations for Healthcare Scale (NEHS) [22] consists of five items which determine the extent to which each participant expects the following negative experiences to occur in their future healthcare encounters: “to be left alone for extended periods of time,” “to be ignored,” “to have your symptoms minimized, “to be judged,” and “to be left out of your treatment planning.” Responses are rated on a five-point Likert scale (0 to 4) ranging from “not at all” to “extremely.” The NEHS items are summed to create an overall score, such that higher scores indicate more negative expectations for future healthcare. This scale has demonstrated good internal consistency in previous studies (e.g., α = 0.84 in over 1000 college students as reported by Gigler et al., 2024) [3]. The expectation items were administered twice, once after the participants read and imagined themselves in the initial HIB encounter and a second time after participants read and imagined themselves receiving their randomly assigned HIB repair condition. The internal consistency for the negative expectancy items in the current study was excellent at time one( α = 0.91) and at time two (α = 0.90).

In the current study, four parallel items were created to assess positive expectations for future healthcare encounters (PEHS). These items were “to be listened to”, “to receive clear explanations and instructions about your condition”, “to be treated by staff who show care/concern/compassion”, and “to be treated by staff who are professional in their work”. Response options ranged from 0 “not at all,” to 4 “extremely.” The positive expectations for future healthcare scale had a Cronbach’s alpha of 0.96 in this sample at both administrations (post-HIB and post-repair), demonstrating excellent internal consistency.

#### 2.3.5. Anticipated Healthcare Avoidance/Disengagement

Anticipated healthcare avoidance/disengagement was measured by two previously published questions assessing anticipated avoidance [9,10]. These were “To what extent do you expect to avoid accessing healthcare services after this experience?” and “To what extent do you expect to delay seeking medical care after this experience?” Response options ranged from “not at all” to “extremely,” with higher scores representing a greater anticipated healthcare avoidance/disengagement. The Cronbach’s alpha for this two-item scale was acceptable at 0.78 in the current sample. The measure of anticipated healthcare avoidance was administered only once, after participants experienced one of the four repair conditions. They were instructed to reflect on the totality of their imagined healthcare experiences (i.e., both the initial HIB healthcare encounter and their randomly assigned repair scenario) when answering these questions.

#### 2.3.6. Demographic Questions

Each participant completed demographic questions describing their gender identity, sexual orientation, race/ethnicity, chronic health condition status, and insurance status. These data are presented in Table 1.

## 3. Results

At the outset, patterns of missing data were examined using Little’s MCAR test [23]. Missing data was missing completely at random (*p* > 0.05) for all scales besides Perceived Repair, *p* = 0.03; Post-Repair HIB, *p* = 0.04; and the Post-Repair Wake Forest Trust Scale, *p* = 0.001. These three measures were located near the end of the survey. Due to the length of the study, some participants quit responding before completing these final questionnaires; however, attrition did not differ by condition. Participants were retained similarly in the healthcare provider apology HIB repair (*n* = 47), healthcare administrator apology HIB repair (*n* = 44), organizational change HIB repair (*n* = 50), or control (no repair) (*n* = 50) conditions. Only data from participants who completed at least 80% of these scales were retained for final analysis. As with other missing data, mean imputation occurred for missing values per scale by participant, for only those who completed 80% of that particular scale. Thus, *n*’s vary slightly across analyses.

### 3.1. Two Healthcare Scenarios

A series of independent sample one-way analyses of variance (ANOVAs) revealed no differences in how realistic participants thought the two initial healthcare encounter scenarios were on a scale of one to five, *F*(1, 187) = 0.00, *p* = 0.99 (headache *M* = 4.12, *SD* = 0.92, abdominal pain *M* = 4.12, SD = 0.80). Participants also rated the two scenarios as equally easy to imagine themselves in and equally as likely to happen to them (*F*’s < 1).

Importantly, the two scenarios were also perceived to contain similar levels of HIB, *F*(1, 189) = 1.14, *p* = 0.29. Moreover, the two initial HIB-containing healthcare encounter vignettes generated similar levels of distrust in the healthcare team, *F*(1, 187) = 0.30, *p* = 0.59, as well as negative, *F*(1, 189) = 0.83, *p* = 0.36, and positive expectations for future healthcare, *F*(1, 189) = 0.93, *p* = 0.34. Given that the two initial healthcare scenarios functioned very similarly, all subsequent analyses combined responses across these two health condition encounters to increase statistical power.

### 3.2. Data Analytic Strategy

IBM SPSS statistics software package 29.0 was utilized for data cleaning and analyses. Prior to conducting the primary analyses, basic statistics (means, standard deviations, and ranges) were computed and the data was inspected for outliers and missing values.

Validity checks were used to confirm that participants perceived their assigned encounter as containing healthcare institutional betrayal after reading the healthcare encounter vignette designed to elicit HIB. It was not expected that four groups (3 experimental repair conditions and one control/no repair) would differ from one another on the dependent variable measures after reading the healthcare institutional betrayal vignette, since exposure to HIB was equivalent for all participants. However, to confirm that there were no group differences at baseline, a one-way ANOVA was conducted on perceived levels of institutional betrayal in the assigned healthcare encounter. As expected, no group differences in levels of HIB were found, *F*(3, 186) = 0.79, *p* = 0.50.

Next, to test the hypothesis that experiencing repair would serve to lessen residual HIB, restore trust in the healthcare team, lessen negative expectations for future healthcare, and increase positive expectations for future healthcare, four separate between-group (4 repair conditions) by within-group repeated measure (pre- to post-repair) general linear models were conducted. For each of the four main outcome measures, the pre-condition or first administration of each measure was after reading and imagining experiencing the healthcare encounter that included HIB. The second administration occurred after participants experienced one of the four repair conditions. A priori, we expected to obtain four main effects for repair as well as four repair group x time of administration interactions. In the interactions, we expected no changes across time in the outcome measure for the no repair group (control) and the greatest changes for the organizational change repair condition. Follow-up ANOVAs were considered to determine whether the pre–post repair change was significant for each experimental Between-Subjects group. Finally, post hoc analyses (Tukey’s Method Least Significant Differences) were used to determine whether the four post-repair means differed significantly between each group for each specific outcome (HIB, trust, negative expectations, positive expectations, anticipated avoidance).

### 3.3. Repair Condition Validity Check Analyses

As expected from the planned manipulation check, results of a one-way ANOVA indicated a significant effect of the HIB repair condition on participants’ perceptions of repair, *F*(3, 182) = 45.36, *p* < 0.001. Tukey’s LSD tests for multiple comparisons found that participants in all three HIB repair conditions reported significantly higher repair scores compared to participants assigned to the no HIB repair control condition (*n* = 47; *M* = 9.91; all *p*’s < 0.001). Mean HIB repair scores were significantly different between the healthcare provider apology (*n* = 47; *M* = 18.98, *SD* = 4.50) and healthcare administrator apology repair conditions (*n* = 43; *M* = 16.32, *SD* = 5.12; 95% CI = 0.68-4.63; *p* = 0.009), with an apology from the healthcare provider generating greater HIB repair than the same repair actions undertaken by a healthcare administrator. Mean repair scores were also significantly different between the organizational change repair condition (*n* = 47, *M* = 20.37, *SD* = 4.16) and the healthcare administrator apology condition, with the organizational change repair condition generating greater perceptions of repair (95% CI = 2.1–6.0; *p* < 0.001). However, contrary to expectation, there were no significant differences in perceptions of repair between the healthcare provider apology and the organizational change HIB repair conditions (*p* = 0.15). The overall effect size of HIB repair condition on repair scores was moderate in strength (η^2^ = 0.42).

### 3.4. Repairing Healthcare Institutional Betrayal

As hypothesized, there was a significant interaction effect (i.e., for the 4 between-group by two within-subject repeated measure ANOVA) on residual HIB scores, *F*(3,185) = 21.46, *p* < 0.001 (see Table 2). This interaction accounted for 26% of the variance in changed HIB scores. There was also a main effect for repair condition on residual HIB, *F*(1,185) = 158.90, *p* < 0.001, η^2^ = 0.46.

Follow-up Univariate ANOVAs for each of the four groups indicated a significant effect of receiving repair on levels of HIB for all three repair groups (*p*’s < 0.001), but not for the control group. Consistent with the obtained interaction effect, both the healthcare provider apology and organizational change repair conditions were highly effective at reducing HIB, accounting for 35% of the HIB score change and 37% of the HIB score change respectively. The healthcare administrator apology condition produced a weaker reduction in HIB, accounting for 8.5% of the change variance. There was no significant reduction in HIB for participants who were randomly assigned to the control condition.

This pattern of results is depicted in Table 2, as Tukey’s Least Significant Difference analyses indicated that the mean residual HIB for all three repair groups was lower than what was reported by participants in the control group. However, group one and group three (Healthcare Provider apology and Organizational Change repair) reported lower mean residual HIB at time two than did group two (Healthcare Administrator apology).

### 3.5. Healthcare Trust

As hypothesized and shown in Table 2, there was also a significant interaction effect on healthcare team trust scores, *F*(3,185) = 13.86, *p* < 0.001. This interaction accounted for 18% of the variance. There was also a main effect for repair, *F*(1,185) = 127.19, *p* < 0.001, *η*^2^ = 0.41.

The pattern of results is similar to what was found and reported above with residual HIB as the outcome measure. Specifically, for the healthcare team trust variable, follow-up Univariate ANOVAs for each of the four groups indicated a significant effect of receiving repair on trust for all three repair groups (*p*’s < 0.001), and also for the control group *(p* = 0.04). Both the healthcare provider apology and organizational change repair conditions were effective at helping to restore trust, accounting for 26% of the trust score change and 33% of the trust score change respectively. The healthcare administrator apology condition produced a weaker increase in trust, accounting for only 4% of the change variance. Repeating the measurement twice accounted for 2% of the change in trust scores for participants who were randomly assigned to the control condition.

With regard to the final post-repair trust scores, pairwise comparisons showed statistically significant differences in mean levels of restored trust between the healthcare provider apology and the organizational change repair conditions versus the healthcare administrator and the control conditions. Healthcare provider apology and healthcare system organizational change repair conditions did not differ significantly from one another in mean restored healthcare-related trust.

### 3.6. Negative and Positive Healthcare Expectations

As hypothesized, there was a significant interaction effect (4 between-group repair conditions by the two within-subject time administrations, once pre- and once post-repair) on changing negative expectations for future healthcare, *F*(3,187) = 5.70, *p* < 0.001 (see Table 2). This interaction accounted for 8.4% of the variance, indicating a small effect size. There was also a main effect for repair on reducing negative expectations for future healthcare, *F*(1,187) = 28.31, *p* < 0.001, η^2^ = 0.13.

The pattern of findings for the negative expectation outcome variable is shown in Table 2. Follow-up Univariate ANOVAs for each of the four groups indicated a significant effect of receiving repair on reducing negative expectations for future healthcare after HIB for only two repair condition groups, the healthcare provider apology HIB repair (*p* < 0.05) and organizational change HIB repair conditions (*p* < 0.001). Participants who were randomly assigned to the organizational change repair condition showed the most reduction in their post-HIB negative expectations, with 17% of their score change explained. While there was also a significant reduction in the negative expectations of those assigned to the healthcare provider apology condition, only 3% of score change variance was explained. Finally, as shown in Table 2, participants randomly assigned to the no HIB repair (control condition) continued to report the most negative expectations for future healthcare encounters and this mean was significantly different from the mean obtained from those randomly assigned to the healthcare provider apology condition. The post-repair negative expectation mean score for the two most effective repair strategies were not found to differ significantly from each other.

As hypothesized, there was also a significant interaction effect (4 between-group repair conditions by two within-subject time administrations, once pre- and once post-repair) on changing positive expectations for future healthcare, *F*(3,187) = 5.78, *p* < 0.001 (see Table 2). This interaction accounted for 8.5% of the variance, indicating a small effect size. There was also a main effect for repair on reducing positive expectations for future healthcare, *F*(1,187) = 26.76, *p* < 0.001, *η*^2^ = 0.13.

The pattern of findings for the positive healthcare expectations outcome variable is shown in Table 2. Follow-up Univariate ANOVAs showed a different pattern than was obtained for negative expectations. Specifically, the ANOVAs indicated a significant effect of receiving repair on increasing positive expectations for future healthcare after HIB for two repair condition groups, the healthcare administrator apology (*p* < 0.001) and organizational change repair conditions (*p* < 0.001). The change in positive expectations for the healthcare provider apology condition was marginally significant (*p* = 0.05) and accounted for only 2% of the variance. Again, participants who were randomly assigned to the organizational change repair condition showed the most improvement in their post-HIB positive expectations, with 13% of their positive expectation score change explained. While there was also a significant increase in the positive expectations of those assigned to the healthcare administrator apology condition, only 6% of change variance was explained.

### 3.7. Avoidance/Disengagement

Anticipated avoidance was only measured once at the end of the survey, so pre-repair avoidance scores could not be controlled for in this analysis. Results from a one-way ANOVA indicated that there was a statistically significant effect of HIB repair condition on reducing residual anticipated healthcare avoidance, *F*(3, 186) = 2.70, *p* < 0.05. The between-groups repair condition effect accounted for 4% of the variance in final anticipated avoidance scores. As shown in Table 2, the pattern of means indicated that the mean anticipated avoidance scores in the healthcare provider apology repair condition were significantly lower than the mean anticipated avoidance scores in the control condition (*p* < 0.05). No other mean differences were significant.

## 4. Discussion

This study examined the perceived effectiveness of two potential HIB repair strategies (empathic apology vs. organizational change) undertaken by different healthcare stakeholders (the healthcare provider and a healthcare system administrator) to repair and mitigate the known consequences of HIB [17,20]. Two consequences to problematic healthcare encounters have been focused on in the HIB literature: reduced trust [3,8] and anticipated avoidance. This study considered a third consequence: changed healthcare expectations, both positive and negative. Changes in residual levels of HIB were also assessed. Repair from HIB is essential given its prevalence among the worst healthcare experiences of college students [3,8], and its direct and indirect associations with deleterious health outcomes. Importantly, this is the first known study to utilize an experimental design to better elucidate the potential restorative effects associated with specific HIB repair strategies undertaken by individuals embedded in the healthcare system.

As hypothesized, participants assigned to any of the three HIB repair conditions reported greater changes in perceived repair compared with those assigned to the no repair control condition, suggesting that any reparative action by healthcare team members or healthcare system representatives is likely to be perceived as restorative. Similarly, all three experimental repair conditions resulted in reductions in perceptions of HIB experienced in the initial healthcare encounter while no changes in residual HIB were seen among those randomly assigned to the no repair control condition. These findings support the assertion that multiple stakeholders within the healthcare system (i.e., healthcare providers and healthcare system administrators) can take action to repair perceptions of healthcare institutional betrayal.

However, contrary to expectation, perceptions of repair did not differ between those assigned to the healthcare provider apology condition and those assigned to the organizational change repair condition. In fact, across all tested HIB impact variables, these two different repair approaches facilitated significant recovery and resulted in mean outcome variable scores that typically did not differ from one another. These findings fail to support hypotheses, generated from the BITTEN model and betrayal trauma theory, that courageous healthcare system organizational change actions would result in greater perceptions of repair, as this commitment by the system might address the individual’s HIB while potentially reducing instances of future HIB for others.

Another outcome variable considered in this study was patient trust in the healthcare team. Given the substantial prevalence of nondisclosure of medically relevant information (61% of an MTurk sample), trust remains critical for high-quality care [24]. Obtained findings suggest that although participants in all conditions, experimental and control, reported significantly increased trust at the second measurement, two pathways were especially effective in restoring healthcare-related trust. Both had moderate effect sizes. Path one is provider-centered in that the healthcare provider directly acknowledges the harm experienced by the patient and offers an apology. The second pathway acknowledges that the harm experienced was institutional in nature, as it requires administrators within the healthcare institution or system to agree to respond to the patient’s HIB through policy and/or procedure changes. Theoretically, this second pathway not only helps the individual post-HIB but also demonstrates institutional courage as the system works to prevent harm and enact quality care for all patients over time.

Previous research has shown that anticipated avoidance of healthcare is a robust consequence of healthcare institutional betrayal [9,10] and addressing this outcome is imperative in young adults. Unfortunately, anticipated avoidance was only measured once, at the end of this study. Thus, we could not control for initial levels of anticipated avoidance, nor could we test for changes in anticipated avoidance by repair condition. Furthermore, these results are based on a two-item measure that, while face valid, may lack the sensitivity to detect differences, particularly since the mean level of anticipated avoidance was relatively low in this college student sample. However, findings did indicate a significant repair condition difference on mean levels of anticipated healthcare avoidance at the study’s conclusion. Lower mean levels of anticipated healthcare avoidance were reported by participants in the healthcare provider apology condition compared to the no repair condition, suggesting potential benefit from this particular repair strategy. However, the effect size for this change was quite small and replication will be essential.

It is possible that young adult participants may find it difficult to re-engage with healthcare systems after experiencing HIB, despite reparative actions. Other factors may also influence anticipated healthcare avoidance among college students including the presence of early maladaptive schema, experiences of racial discrimination [10], lack of healthcare literacy, or a general belief in their health invincibility. Anticipating avoidance or delaying future healthcare engagement might also be conceptually more resistant to short-term repair interventions than other outcomes like trust, residual HIB, or future healthcare expectations. A supportive trusting relationship with a consistent provider may facilitate engagement. This latter explanation is partially bolstered by the finding that the lowest mean anticipated avoidance was in the healthcare provider apology condition. Furthermore, previous research indicates that increased provider trust is a mediator between HIB and anticipated healthcare avoidance. Additional research is needed to explicate these relationships and further examine the possible ways to repair anticipated healthcare avoidance, in a variety of samples.

This study considered an additional, novel impact that may occur after experiencing HIB in a healthcare encounter: changed expectations for future healthcare encounters. While overall results indicated that experiencing repair was effective in addressing negative and positive expectations that were impacted as a result of HIB, the effect sizes for changing future expectations were generally smaller than those obtained with other outcomes (i.e., changing residual HIB and increasing perceptions of trust). Again, it may take more consistent repair to change future expectations and behavior.

Furthermore, interesting change patterns emerged in these analyses that were consistent with a priori expectations about the importance of organizational change. For example, although two repair conditions resulted in significant reductions in negative expectations (i.e., organizational change and healthcare provider apology); the eta squared for the organizational change condition was substantially larger than for the healthcare provider apology condition (17% versus 3%). Elevated negative expectations remained in the healthcare administrator apology condition suggesting that this repair strategy is not particularly effective in reducing future skepticism about healthcare. The courageous act of organizational change may be a particularly important way to repair future-orientation harm.

Meanwhile, when working to restore positive healthcare expectations following a HIB-laden healthcare encounter, all three repair strategies were helpful, as participants within any of three HIB repair conditions reported greater positive expectations for future healthcare encounters after receiving repair. However, the amount of variance accounted for by experimental conditions differed. The lowest amount of change variance occurred in the healthcare provider apology condition (2% of the change variance accounted for), although this group ended with the highest levels of positive expectations for healthcare. The greatest difference in pre- to post-positive expectation scores occurred in the organizational change condition, accounting for 13% of the variance, compared to 6% in the healthcare administrator apology condition. This result further supports the importance of organizational change as a repair strategy. Replication of these findings will be essential.

In summary, this work was theoretically informed by betrayal trauma theory [25] and the BITTEN model of trauma-informed care [14]. Lewis and colleagues (2019) [14] posited that there is a need for patients’ current healthcare providers to initiate repair following HIB (regardless of whether they were involved in the original HIB) in order to improve the patient’s interaction with the healthcare system in the future. This study affirms that acts of repair can address residual HIB effects. All three repair strategies showed the ability to facilitate restored healthcare provider, team, and system trust. In keeping with the suppositions of the BITTEN model, repair, at the interpersonal-provider level and at the organizational change level, appear to be two critical methods to address patient responses to HIB as well as two potential pathways toward helping patients heal their relationship with the healthcare system. Study results map onto a large body of work indicating that patients’ positive experience of healthcare is related to treatment adherence and can serve to enhance the quality of care [26]. Providers continue to play a large role in the patient experience as they can facilitate optimal communication of patient concerns, support shared decision-making, and co-develop a clear, responsive, and individualized treatment plan [27]. However, there is room for action from other members of the healthcare team as well as from healthcare system administrators. In fact, changing future expectations for healthcare may require policy and procedure changes, enacted organizationally.

### 4.1. Practice Implications

Importantly, these findings also indicate that a short, targeted empathic acknowledgement and apology, often by the patient’s healthcare provider, an act that takes relatively little time and no money, holds the potential to facilitate clinically meaningful repair between an individual and their healthcare system. As such, it may be important to consider training healthcare professionals to routinely assess for, recognize, and respond to HIB as a standard part of patient care, as providers are unlikely to address HIB they are unaware of. This training could be provided to all patient-facing medical staff. However, this strategy may not be effective at preventing subsequent HIB, as systemic failures will likely persist if left unaddressed. Therefore, efforts to encourage organizational courage on the part of healthcare systems remain a necessity.

### 4.2. Study Limitations and Future Directions

One limitation of this study was that the sample was composed entirely of young adult (under 30 years old) college students who were imagining themselves experiencing a routine healthcare encounter that contained multiple acts of HIB. Results may not generalize to other patient populations even though researchers have found that college students already had significant interactions with the healthcare system and their past problematic interactions often included HIB [3]. Findings are also bolstered by participants’ assessment that the encounter they read was realistic, seemed like something that could happen to them, and was easy to imagine experiencing. A second limitation was that data were collected cross-sectionally using self-report instruments. Shared method variance and participant fatigue were methodological concerns. Longitudinal studies and observational designs would benefit the field.

Because the opening HIB scenario constituted only one negative healthcare encounter, this study’s results may also not generalize to patients with chronic or ongoing healthcare situations that often contain HIB. Future studies may consider testing whether differing lengths of experiencing HIB (one-time vs. chronic) influence participants’ perceptions of repair strategies. For example, the organizational change repair condition may be more important when repairing long-standing or repetitive HIB encounters occurring among patients with chronic conditions, or HIB that seems to be differentially directed to patients from vulnerable populations. Furthermore, continued efforts are needed to study HIB from a population-health perspective and to determine whether organizational change is critical for sustainable HIB reduction and as a way to address perceptions of the broader system.

Several other future directions emerged from this study. First, future research needs to explore how to train professionals to systematically and efficiently assess patients for prior HIB that could be activated in the current encounter. This type of screening may be integrated into encounters, much like the PHQ-9 is now routinely delivered to assess behavioral health needs. Moreover, future studies on HIB in general, and on healthcare institutional betrayal repair in particular, should focus on recruiting historically underserved populations to these studies, given the breadth of literature indicating that those with minority identities are more likely to experience HIB [4,9,28] and these participants may react differently to individualized apologies versus systemic change. Qualitative research could help determine whether certain repair strategies have already been experienced by patients but were not perceived as repairing; whether the timing of a repair strategy impacts perceived repair; whether there are longer-term impacts of specific repair behaviors on patient health; and even the degree to which patients view their healthcare provider as a key representative of the larger system and as acting on behalf of the organization as a whole, or not. Future studies may consider the chronicity or severity of the illness or condition that was occurring during HIB exposure and whether this HIB is associated with the current provider or is activated in the current encounter as these variations likely impact encounter perceptions as well as the utility of certain HIB repair strategies.

Finally, although healthcare provider HIB repair, via an empathic acknowledgement and apology, was effective in improving trust, increasing positive future healthcare expectations, and reducing HIB, there are many implementation barriers to consider for this strategy. For example, physicians have historically been advised against making apologies or admitting to medical errors to avoid malpractice lawsuits [29]. Although apology laws, which allow physicians to apologize without those statements being used in lawsuits [30], have made this kind of practice more acceptable, some research suggests that professional norms in the field of medicine still preclude apologies. Finally, apology laws have not been shown to effectively facilitate physician transparency [31,32]. There are also issues with healthcare provider time and capacity to recognize and address HIB; both resources can be in short supply within a given healthcare encounter.

## 5. Conclusions

Findings indicated that various reparative actions, undertaken by specific stakeholders, significantly mitigated some negative outcomes associated with HIB (e.g., reducing residual HIB, residual trust deficits, lessening negative future expectations, reducing anticipated avoidance, and increasing positive expectations for future healthcare). As such, a healthcare experience containing HIB does not (necessarily) damage the patient–provider–healthcare system relationship irreparably. Further, this study indicates that there are specific actions that various stakeholders can take to facilitate patient recovery post-HIB. Having a healthcare provider acknowledge and apologize to patients who have experienced HIB results in significant repair and may be a critical first step as the institution gathers its courage to enact more cost-prohibitive and time-intensive organizational changes.

## Figures and Tables

**Table 1:** Demographic variable frequencies.

Variables	*N*	%
**Gender Identity**		
Man	76	39.8%
Woman	109	57.1%
Transgender or Genderqueer	6	3.0%
**Sexual Orientation**		
Heterosexual	149	78.8%
Gay/Lesbian	7	3.5%
Bisexual	22	11.6%
Queer	1	0.5%
Questioning/Unsure	6	3.2%
Pansexual	4	2.1%
Prefer not to respond or missing	2	1.0%
**Race**		
White	98	51.3%
Black/African American	38	19.9%
South or East Asian	17	8.9%
Indigenous, Native or First World	3	1.6%
Multiracial	17	8.9%
Latino or Hispanic Self-Identified	9	4.7%
Prefer not to respond	9	4.7%
**Chronic Health Condition**		
Yes	28	14.7%
No	156	81.7%
Prefer not to respond	7	3.7%
**Health Insurance Status**		
Uninsured	3	1.6%
Medicaid	40	20.9%
Private Insurance	123	64.4%
Prefer not to respond	25	13.1%

**Table 2:** Changes in residual HIB, trust, avoidance, negative, and positive expectations across time by repair condition.

Variable	Healthcare Provider Apology HIB Repair *N* = 47 *M(SE)*	Healthcare Administrator Apology HIB Repair *N* = 44 *M(SE)*	Organization Change HIB Repair *N* = 50 *M(SE)*	No HIB Repair Control *N* = 50 *M(SE)*
Pre-Repair (Post-HIB) Institutional Betrayal	152.90 (4.93)	143.53 (5.04)	152.71 (4.73)	147.88 (4.78)
SIG CHANGE	YES *p* < 0.001	YES *p* < 0.001	YES *p* < 0.001	NO
Post-Repair Institutional Betrayal	111.27 (4.81) ^a^	125.63 (4.92) ^b^	110.62 (4.62) ^a^	144.65 (4.66) ^c^
Pre-Repair Trust	24.05 (1.17)	25.34 (1.75)	22.06 (1.66)	24.66 (1.64)
SIG CHANGE	YES *p* < 0.001	YES *p* < 0.004	YES *p* < 0.001	YES *p* = 0.05
Post-Repair Trust	34.87 (10.58) ^a^	28.78 (10.72) ^b^	34.38 (8.89) ^a^	27.45 (11.82) ^b^
Pre-Repair Expect-NEG	5.14 (0.92)	6.95 (0.93)	9.05 (0.89)	7.21 (0.89)
SIG CHANGE	YES *p* < 0.05	NO	YES *p* < 0.001	NO
Post-Repair Expect-NEG	3.58 (0.75) ^a^	5.77 (0.78) ^b^	4.81 (0.73) ^a,b^	6.72 (0.73) ^b^
Pre-Repair Expect-POS	12.32 (0.81)	10.21 (0.84)	9.32 (0.79)	10.98 (0.79)
SIG CHANGE	YES *p* < 0.05	YES *p* < 0.001	YES *p* < 0.001	NO
Post-Repair Expect-POS	13.49 (0.62) ^a^	12.34 (0.64) ^a,b^	12.36 (0.61) ^a,b^	10.76 (0.60) ^b^
Post-Repair Avoidance	3.91 (1.98) ^a^	4.55 (1.91) ^a,b^	4.66 (2.00) ^a,b^	5.02 (1.87) ^b^

Note. Post-HIB encounter scores were collected prior to participants being randomly assigned to the repair experience for all variables except avoidance. Anticipated avoidance was only assessed once. Post-HIB healthcare encounter scores are time one in the repeated measure ANOVAs. Time two scores were collected after the repair vignette. Ns vary slightly across analyses due to missing data. Post-Repair means with different superscripts are significantly different from one another using Tukey’s Least Significant Differences. Means in the same row that share the same letter superscript, are not significantly different from one another. Significant changes in scores from pre-repair to post-repair for each measure are shown in the Significant Change rows.

## Data Availability

Data are available upon reasonable request.
